# Unveiling perceptions on academic leadership effectiveness: PLS-SEM, FSQCA, and NCA approaches

**DOI:** 10.1371/journal.pone.0320723

**Published:** 2025-04-14

**Authors:** S. M. Mahbubur Rahman, Ummah Tafsirun, Md. Faisal-E-Alam, Paulo Ferreira, Luís Loures, Rui Alexandre Castanho

**Affiliations:** 1 Department of Business Administration, Noakhali Science & Technology University, Noakhali, Bangladesh; 2 Department of Management Studies, Begum Rokeya University, Rangpur, Bangladesh; 3 Portalegre Polytechnic University, Portalegre, Portugal; 4 CEFAGE, IIFA—Center for Advanced Studies in Management and Economics, Universidade de Évora, Palácio do Vimioso, Largo Marquês de Marialva n, Évora, Portugal; 5 VALORIZA—Research Centre for Endogenous Resource Valorization, Portalegre Polytechnic University, Campus Politécnico, Portalegre, Portugal; 6 Faculty of Applied Sciences, WSB University, Dabrowa Górnicza, Poland; 7 Advanced Research Centre, European University of Lefke, Mersin, Turkey; 8 College of Business and Economics, University of Johannesburg, Johannesburg, South Africa; East China Normal University, CHINA

## Abstract

Academic leadership plays a critical role in fulfilling higher education institutions’ missions, fostering a competent workforce, and becoming a key driver in unlocking the potential to achieve sustainability goals. Within this context, the leadership effectiveness of academic deans is particularly significant. This study aims to identify the factors influencing the academic leadership effectiveness in public universities in Bangladesh. The study collected data from 318 faculty members of public universities. A combined methodology consisting of Partial Least Squares-Structural Equation Modeling (PLS-SEM), Fuzzy-Set Qualitative Comparative Analysis (fsQCA), and Necessary Condition Analysis (NCA) was utilized to analyze the collected data. The PLS-SEM results indicated that vision and goal setting (VG), management of the unit (MU), interpersonal relationships (IR), communication skills (CS), research/professional endeavors (PE), and quality of education in the unit (QEU) significantly influence deans’ leadership effectiveness. Further, five necessary and six sufficient conditions were discovered by fsQCA results, which also demonstrated the nonlinear and intricate interaction effects of the factors leading to leadership effectiveness (LE). Importantly, NCA findings revealed that all factors are essential for LE and have meaningful and substantial impact. Also, a minimum of 14.78% VG, 20.75% MU, 23.27% IR, 33.96% CS, 16.98% PE, and 10.06% QEU must be met to accomplish an 80% LE. Therefore, the findings provide useful insights for the higher education sector, university top management, potential academic leaders, and relevant stakeholders to improve leadership effectiveness at the tertiary education level. Moreover, this study is the first to explain the effectiveness of deans’ leadership through an expanded methodology that integrates both symmetric and asymmetric methods.

## 1. Introduction

In this contemporary world, leadership at the tertiary education level has assumed a crucial role in advancing the quality of higher education [1]. The overarching goals of higher education institutions—academic excellence, innovation, and positive societal impact—are guided by their leaders [2]. As the diversity of universities continues to rise and the policy reforms in numerous countries reinforce the strategic role of academic leaders, academic leadership is rising rapidly to the forefront of scholarly attention [3]. In recent years, effective academic leadership has become increasingly critical in higher education. This is especially true in developing nations like Bangladesh, where challenges such as resource constraints, global competition, and policy reforms significantly impact institutional success [4].

The recent World University Rankings 2024, published by Times Higher Education (THE) [5], recognized only four Bangladeshi universities that placed in the 800-1000 category, of which only two are public universities. In terms of Asia rankings for 2024, three public universities in Bangladesh are ranked between 300 and 400. When considering the other emerging SAARC countries, 43 Indian universities and 19 Pakistani universities have secured positions in the 1000 range in this global ranking list. Furthermore, 18 Indian universities and 3 Pakistani universities were ranked in the top 200 in Asia. The disparity in rankings between Bangladeshi universities and their regional counterparts emphasizes the urgent need for strong leadership to improve the country’s educational quality and reputation [6]. Effective academic leadership can ensure academic quality [7]. Research on effective leadership in higher education also shows that leaders and leadership sharpen higher education institutions’ performance, governance, learning, and teaching [8–10].

Earlier research investigated academic leadership effectiveness from multiple viewpoints. Mishra et al. [11] investigated the essential components of academic leadership development and the qualities of effective academic leadership. Yasin et al. [12] examined the expected qualities of an effective academic leader and the perceived effective academic leadership and concluded that an effective academic leader should possess IQ and EQ, visionary power, and the ability to unite their team. Ambag et al. [13] found that academic leaders are transformational and demonstrate strong communication and visioning skills. While academic leadership has been extensively studied, most research has focused on institutions in Western or economically developed nations [14–16].

To date, research on academic leadership effectiveness in developing nations, particularly in the context of Bangladesh’s public universities, is limited. Few studies have specifically examined the distribution and inequality of gender in academic leadership positions in university settings [17,18]. This study aims to fill this gap by exploring academic leadership effectiveness in Bangladeshi public universities. The main aim of this study is to examine the factors that shape the perceptions of deans’ leadership effectiveness in public universities in Bangladesh. The research question is: What factors contribute to deans’ leadership effectiveness in public universities in Bangladesh?

Undertaking this research provides knowledge that can help policymakers and university leaders develop strategies to enhance academic leadership. Understanding these perceptions is vital for creating and encouraging a productive academic environment. In addition, this research employs a hybrid methodology that has been overlooked in prior research. To the best of the authors’ knowledge, this is the first study to use a hybrid methodology combining PLS-SEM, fsQCA, and NCA in the domain of academic leadership. The remaining sections of the paper are organized as follows: Section 2 provides a literature review to support hypothesis development and presents the conceptual framework. Section 3 explains the study’s methodology. Section 4 shows the results of the PLS-SEM, fsQCA, and NCA. Section 5 discusses the main results and theoretical and managerial implications of the findings. Finally, Section 6 covers the conclusions and limitations of this study, as well as future research scopes.

## 2. Literature review and hypothesis development

### 2.1. Academic leadership

Yukl [19] defines leadership as “the process that involves guiding individual and collective efforts to understand and influence people, helping them recognize what needs to be done and how to achieve shared objectives.” Academic leadership is gaining prominence as universities become increasingly complex. Policy reforms in many countries are also reinforcing the strategic role of university leaders. A challenge in the definition of academic leadership is the recognition of the variations in roles and titles that exist among institutions [20]. According to the literature, academic leaders can assume formal leadership roles, such as directors, supervisors, and deans, or informal roles, such as faculty members [21]. With their own unique challenges, there is a growing recognition of the significance of leadership across all levels of an organization, including deans, department chairs, and curriculum and course directors [3]. Conventional knowledge about academic leadership concentrates on the number of responsibilities or actions executed by each individual [22]. In this regard, academic leadership is defined as a collection of duties that include visionary planning, research, and teaching [22]. However, several scholars argue that this particular conceptual framework is unsuitable for the present-day context of higher education. This is primarily because leadership in the contemporary academic arena is not an outcome or responsibility of an individual but rather a group effort [23].

Wolverton and Gmelch [24] offered a comprehensive examination of the leadership exhibited by college deans. Following an extensive review of the relevant literature, they defined academic leadership in American higher education as empowering faculty and staff to establish a community of scholars to achieve common objectives and set direction. Recent years have witnessed an explosion of studies about leadership in academic context, which has led to a diverse view of academic leadership. Siddique [25] posits academic leadership as the leadership type needed in the higher education institution. This term is usually used to describe leadership inside an academic organization and interactions with academic personnel [3]. It differs from professional managers and administrators within universities, who are responsible for numerous non or semi-academic areas of student and research activities. Academic leadership is also referred to as leading academic professionals in teaching, research, and service. Therefore, Dopson [26] urged for additional investigations to establish a thorough definition of leadership in academic environment, encompassing leadership processes more autonomously and contextually while expanding the scope of individual leaders.

### 2.2. Academic leadership effectiveness

The roles of deans and directors are crucial to the success of their respective institutions as academic leaders. According to Morris [27], an academic dean acts as the head of a college or school at a university, whether public or private. The dean administers the university’s faculty and serves as the institution’s leader. They are deemed exceptional due to their more challenging, deliberate, sophisticated, and managerial duties [28]. Tahsildar [29] reported a strong positive correlation between the deans and the faculty members’ levels of efficacy in leadership, teaching, and scholarly work. A study by Hunde et al. [30] aimed to examine the effectiveness of deanship and identify that instructors with different educational qualifications and work experiences had similar views on the effectiveness of deanship in their colleges. Akbulut et al. [31] identified the faculty perceptions of department chairs’ leadership effectiveness and found that the most influential factor was establishing leadership functions, including roles as motivator, visionary, and innovator.

Rosser et al. [32] looked at the leadership of academic deans and directors from both personal and institutional perspectives, focusing on seven key leadership duties. This encompasses (a) vision and goal setting, (b) management of the unit, (c) interpersonal relationships, (d) communication skills, (e) quality of unit’s education, (f) research, professional, and community endeavors, and (g) support for institutional diversity. In empirical research, academic leadership has received limited attention beyond theoretical investigations [33]. Heck et al. [34] identified seven deans’ leadership domains based on past studies and concepts. Rosser et al. [32] used those parameters to evaluate deans and directors. The relevance of these domains has been validated in scholarly investigations concerning leadership in the administration of higher education [35–38]. Hunde et al. [30] also assessed deans’ leadership using these dimensions. Hence, this study adopts these validated leadership dimensions to accurately assess leadership effectiveness in the context of higher education in emerging country.

### 2.3. Theoretical foundations in academic leadership

Academic leadership effectiveness has increasingly been examined through well-established theories in organizational and educational leadership, including transformational, relational, situational, contingency leadership theories, and agile leadership theory. Transformational leadership theory, developed by Burns [39] and later expanded by Bass [40], emphasizes leaders’ abilities to inspire a shared vision, set strategic goals, and motivate followers toward organizational objectives. This approach highlights the importance of constructs such as Vision and Goal Setting, Interpersonal Relationships, and Research/Professional Endeavors in measuring leadership effectiveness, as these traits foster organizational alignment, commitment, and a culture of scholarly excellence.

Relational leadership theory, established by Uhl-Bien [41], further supports the significance of Interpersonal Relationships and Communication Skills by emphasizing the role of interpersonal dynamics and open communication in fostering collaboration and cohesion within academic institutions. Effective relational leadership in academia helps build a supportive environment, enhancing both faculty satisfaction and organizational commitment.

Situational Leadership Theory provides a foundation for Management of the Unit by suggesting that effective leaders adapt their management style according to the needs of their team and context, balancing direction with support based on situational demands [42]. In academic settings, leaders who effectively manage resources, delegate tasks, and respond to departmental challenges create a stable and productive environment essential for meeting both faculty and administrative needs.

Contingency Theory, introduced by Fiedler [43], supports both Management of the Unit and Quality of Education in the Unit by proposing that a leader’s effectiveness is contingent on how well their management approach aligns with organizational needs and goals. Academic leaders who excel in unit management and emphasize educational quality are better positioned to align resources, faculty, and educational objectives. This alignment directly supports curriculum development, program accreditation, and student success, which are critical to the institution’s overall reputation and performance.

Agile Leadership Theory, as an approach to leadership, emphasizes flexibility, collaboration, and continuous improvement, which are crucial for academic leadership effectiveness [44]. By fostering an adaptive leadership style, agile leaders can dynamically adjust their strategies in response to the changing needs of their teams and the academic environment [45]. This theory supports the development of Vision and Goal Setting, Management of the Unit, and Interpersonal Relationships by prioritizing responsiveness and teamwork. Academic leaders who adopt agile principles are better positioned to create a high-performing, innovative environment that enhances faculty engagement and student success, thereby driving overall leadership effectiveness in academic settings.

In our study, Vision and Goal Setting, Management of the Unit, Interpersonal Relationships, Communication Skills, Research/Professional Endeavors, and Quality of Education in the Unit represent key dimensions validated in previous research on academic leadership effectiveness. These constructs were selected based on their demonstrated relevance in enhancing educational quality, faculty engagement, and organizational governance.

### 2.4. Complexity theory

Complexity theory, originally conceptualized by Warren Weaver [46], provides a foundational framework for this study, addressing the need to understand how interconnected elements interact dynamically in complex systems like academic leadership. In academic environments, leadership effectiveness depends on various interdependent factors—such as vision, resource management, and interpersonal relationships—that interact in non-linear ways. Complexity Theory supports the use of Partial Least Squares-Structural Equation Modeling (PLS-SEM), which is ideal for analyzing these interrelationships among latent variables, offering insights into both direct and indirect effects that simpler models cannot capture [47,48]. Recent studies demonstrate the efficacy of Complexity Theory and PLS-SEM in capturing the multifaceted nature of academic and organizational systems, particularly where multiple factors collectively contribute to outcomes [49].

In line with Complexity Theory’s principles, Fuzzy-Set Qualitative Comparative Analysis (fsQCA) and Necessary Condition Analysis (NCA) enable this study to explore diverse combinations and baseline conditions within academic leadership. fsQCA aligns with the concept of equifinality, where different pathways lead to effective outcomes. This approach is essential in academic settings, where diverse leadership approaches may all lead to similar success based on contextual factors [50]. NCA complements this by identifying conditions that are necessary but not sufficient alone, highlighting the foundational elements that must be in place for leadership effectiveness. Together, these methods reveal both the configurations and essential conditions required for success in complex academic systems, advancing a nuanced understanding of leadership through Complexity Theory [51].

### 2.5. Vision and goal setting

Having a vision and direction to be effective as an academic leader is proven by prior researchers [8,52,53]. Without exception, articulating a clear and compelling vision is a crucial competency for academic deans to become successful leaders. Successful coordination of followers’ endeavors toward fulfilling organizational objectives is contingent upon leaders’ ability to communicate their visions. By articulating a compelling vision and establishing specific goals that are consistent with the changing requirements of students, faculty, and stakeholders, effective leaders in this domain provide strategic guidance [54]. From this point of view, an effective leader must explicitly link a vision to members and stakeholders to gain their support. Scott et al. [33] delineated three primary characteristics that exemplify the essence of effective academic leadership: (a) the act of engaging people in the change process, (b) a specific set of traits or skills, and (c) a particular group of individuals responsible for running a university or unit. Based on these characteristics, academic leadership involves creating a supportive environment for academic success, fostering a shared academic identity and values, and representing collaborative efforts for cross-border partnerships. Thus, the following hypothesis can be drawn from the earlier argument:

**Hypothesis 1 (H1)**: Vision and goal setting positively influence leadership effectiveness.

### 2.6. Management of the unit

When a unit is well-managed, it ensures that resources are properly allocated, goals are clearly defined, and team members are aligned with the academic mission. This creates an environment where academic leaders can focus on strategic initiatives, foster collaboration, and drive innovation. As a result, strong management practices contribute to the overall effectiveness of academic leadership, enabling leaders to successfully guide their institutions toward achieving educational excellence. Jones and Rudd [55] emphasized that the sustainability and effectiveness of change management in higher education institutions greatly rely on leadership. This perspective by Zhu et al. [53] implied that possessing excellent management skills is essential for demonstrating quality leadership. Academic leadership involves a variety of positions and designations within higher education institutions. It spans from task-oriented administrative management to visionary leadership with transformative potential. Additionally, it includes tactical management centered on achieving specific objectives [56]. Based on the arguments presented above, we can derive the next hypothesis:

**Hypothesis 2 (H**_**2**_): Management of the unit positively influences leadership effectiveness.

### 2.7. Interpersonal relationships

Academic leaders must act as coalition builders, negotiators, and facilitators [32]. In terms of interpersonal relationships, academic leaders play a vital role in creating a positive and welcoming atmosphere for both faculty and students within the organization. Researchers evaluated leadership effectiveness based on the impact of leaders on individuals, groups, or organizations [57]. Leaders with strong interpersonal skills can enhance their effectiveness by improving communication and resolving conflicts, which is critical in academic leadership [58,59]. Thus, the effectiveness of an academic leader, according to Avolio and Bass [60], is assessed based on the degree to which leaders fulfill the requirements and anticipations of personnel, including supervisors, followers, and peers. This entails their commitment to implementing their vision and guidance, as well as how much they are liked, respected, and admired by their subordinates. Evans [61] discussed that effective academic leadership necessitates three qualities, with interpersonal relationships being the most significant. Therefore, the hypothesis can be stated as follows:

**Hypothesis 3 (H**_**3**_): Interpersonal relationships positively influence leadership effectiveness.

### 2.8. Communication skills

It is well-known that strong communication skills are essential for effective leadership in many fields, including academia. Academic leaders must effectively communicate in public forums, such as meetings, conferences, and university events, to represent the institution and advocate for its interests. Many studies have examined academic leaders’ abilities and effective qualities. Spendlove [62] suggested that effective institutional-level leadership requires scientific credibility, university experience, administrative abilities, communication, and negotiation skills. Consequently, effective communication skills are crucial for academic leaders, enabling them to lead more effectively and achieve better outcomes in their institutions. Considering the earlier literature and studies [32,34], the following hypothesis can be taken:

**Hypothesis 4 (H**_**4**_**):** Communication skills positively influence leadership effectiveness.

### 2.9. Research/professional endeavors

Academic leaders who are committed to research engage in a variety of scholarly endeavors, such as publishing, conducting research, obtaining grants, and contributing to the intellectual discussion in their respective disciplines. Research has shown that academic leaders who uphold rigorous research agendas enjoy greater visibility, influence, and credibility within their respective institutions [63]. Additionally, professional networks also give members access to resources, support, and best practices, all of which make leadership more effective [64]. Involvement in research and professional networks positively impacts leadership effectiveness by providing leaders with essential resources, knowledge, and collaborative opportunities, thereby enhancing their ability to lead and innovate [32,34]. As a result, the following hypothesis can be suggested:

**Hypothesis 5 (H**_**5**_**):** Research/Professional endeavors positively influence leadership effectiveness.

### 2.10. Quality of the unit’s education

A faculty’s quality of education is determined by a multitude of components, such as instructional methodologies, curriculum development, student support services, and academic achievements. An effective approach, stimulating curricula, knowledgeable faculty, and nurturing learning environments that promote student achievement are all the components of a high-quality education [65]. According to Shahmandi et al. [66], the effectiveness of academic leadership would decline if leaders lacked the necessary knowledge, skills, and behaviors essential for guiding their institutions effectively. Consequently, Hoppe [67] emphasized the importance of defining an effective academic leader profile to help current leaders enhance their abilities, prevent unsuitable selections, and develop future leaders. The quality of education provided by faculty and the effectiveness of the dean’s leadership are closely connected. Faculty members often enhance the quality of education within their departments through their effective leadership [8]. Thus, the preceding discussion leads to the following hypothesis:

**Hypothesis 6 (H**_**6**_**):** Quality of education in the unit positively influences leadership effectiveness.

**Fig 1:**
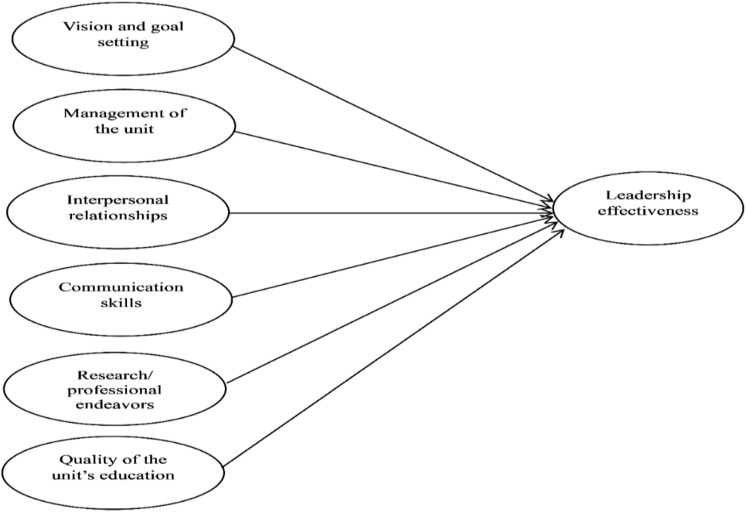
Conceptual model.

Based on the earlier reviews, Fig 1 illustrates the study’s conceptual framework, which includes six independent variables and one dependent variable.

## 3. Materials and methods

### 3.1. Participants and procedures

The current study used a survey questionnaire to validate the conceptual framework. According to Yukl [68], how followers perceive and feel about their leaders is a standard measure of a leader’s effectiveness. Faculty members from all public universities in Bangladesh were targeted as participants. Data collection was conducted from March 1, 2023, to June 30, 2023, at eight purposively selected universities—four general universities and four science and technology universities—to ensure diversity in educational contexts.

A stratified random sampling technique was used, where universities were first categorized by type (general and science/technology), and then faculty members were randomly selected from faculty lists obtained from the universities’ official websites. Participants received an email with a study summary and a link to a Google Form. A total of 318 valid responses were collected, with all participants providing voluntary consent to participate in the study. Table 1 presents the demographic details of the study participants.

**Table 1:** Respondents’ demographic profile (N =  318).

Demographic characteristics	Segments	Frequency	Valid Percent
**Gender**	Male	207	65.1%
	Female	111	34.9%
**Age**	25–30 years	153	48.1%
	31–35 years	105	33%
	36–40 years	43	13.5%
	41 years – above	17	5.3%
**Educational Qualification**	Masters	227	71.4%
	MPhil	44	13.8%
	PhD	37	11.6%
	Postdoc	9	2.8%
	Others	1	0.3%
**Designation**	Lecturer	159	50%
	Assistant Professor	113	35.5%
	Associate Professor	32	10.1%
	Professor	14	4.4%
**Work Experience**	1–5 years	175	55%
	6–10 years	94	29.6%
	11–15 years	38	11.9%
	16 years – above	11	3.5%

Table 1 demonstrates that the majority of respondents, representing 65.1% of the total, were male, while 34.9% were female. Most respondents, 153 (48.1%), were aged between 25 and 30 years, with 105 (33%) falling between the 31 to 36 age categories, while 13.5% belonged to the 36 to 40 years category, and 5.3% were beyond 40 years old. When considering the educational level of the samples, there were 227 Master’s degree holders (71.4%), 44 MPhil holders (13.8%), 37 PhD holders (11.6%), and 9 Postdoc holders (2.8%). Almost half of the participants (50%) identified themselves as lecturers, whereas only 4.4% were professors. Over half of the participants (55%) reported 1 to 5 years of job experience at that university, while 29.6% reported having 6 to 10 years of work experience.

### 3.2. Measurement instruments

The research adopted instruments drawn from existing literature in this domain, and content validity assessed through a pilot study. A structured questionnaire was then developed to collect data from the participants. The study instrument was adapted from Heck et al. [34]. Deans’ effectiveness was assessed using six dimensions, including vision and goal setting, management of the unit, interpersonal relationships, communication skills, research/professional endeavors, and quality of the unit’s education. There were 43 items covering these six independent constructs. Leadership effectiveness construct was evaluated with five items from Hooijberg et al. [69]. All 48 items were rated on a 5-point Likert scale, where a rating of “1” reflected as very unsatisfactory, while a rating of “5” denoted an outstanding level of performance.

### 3.3. Data analysis techniques

The study used three statistical techniques to analyze the collected data. First, Partial Least Squares Structural Equation Modeling (PLS-SEM) was employed as the primary analysis technique using SmartPLS (V. 4.1). PLS-SEM has advanced more rapidly than Covariance-Based SEM (CB-SEM) in recent years and is particularly well-suited for analyzing complex interactions between observed and latent variables. Furthermore, without making any assumptions about distributions, PLS-SEM can estimate complicated models with several constructs, measures, and structural paths. The analysis was conducted in two stages: evaluating the measurement model and the structural model [70].

Second, Fuzzy-set Qualitative Comparative Analysis (fsQCA) is used to augment the results of PLS-SEM and offer a more sophisticated insight into the factors that influence academics’ leadership effectiveness. The investigation of complex causal configurations is facilitated by fsQCA, which is particularly appropriate for samples that are small to medium in size [71]. In addition to providing a qualitative magnitude that outperforms conventional regression-based method, it assists in the identification of necessary and sufficient conditions for an outcome. However, fsQCA advances the investigation by unveiling configurational patterns that conventional linear model may fail to detect [72].

The final method applied is Necessary Condition Analysis (NCA), which adds to the fsQCA approach. NCA proves especially beneficial in better understanding of the configurations that result in high or low level of outcome, as it is linked to the identification of necessary condition for an outcome [73]. By implementing NCA, this study attempts to identify the crucial conditions that must exist for the anticipated results to be achieved. This approach offers additional insights into the complexities of the relationships being examined [74].

## 4. Results

### 4.1. PLS-SEM results

#### 4.1.1. Assessment of the measurement model.

In the PLS-SEM findings, it is crucial to determine whether the measurement model is reflective or formative [75]. In this study, the measurement model is reflective which consists of three key components that need to be considered to ensure reliability and validity during evaluation. First, internal consistency reliability is confirmed by using metrics like Cronbach’s Alpha, Composite Reliability (CR), and rho A. Second, convergent validity was checked with loadings and average variance extracted (AVE) values. Thirdly, discriminant validity was verified [76,77]. The internal consistency reliability measures must meet a minimum threshold of 0.7 [78,76]. It is also recommended that the loadings and AVE values should be greater than 0.5 [79,80]. Fornell-Larcker criterion is a conservative and frequently used metric in the evaluation of discriminant validity [75]. In addition, Heterotrait-monotrait ratio (HTMT) was recommended by Henseler et al. [81] as a way to ensure more robustness of discriminant validity. For the HTMT confidence interval, 1 must not be the threshold. A lower, more conservative threshold value of 0.85 seems justified [81]. In the Fornell-Larcker criterion, the square root of each construct’s AVE should exceed its maximum correlation with every other construct in the same model [82].

**Fig 2:**
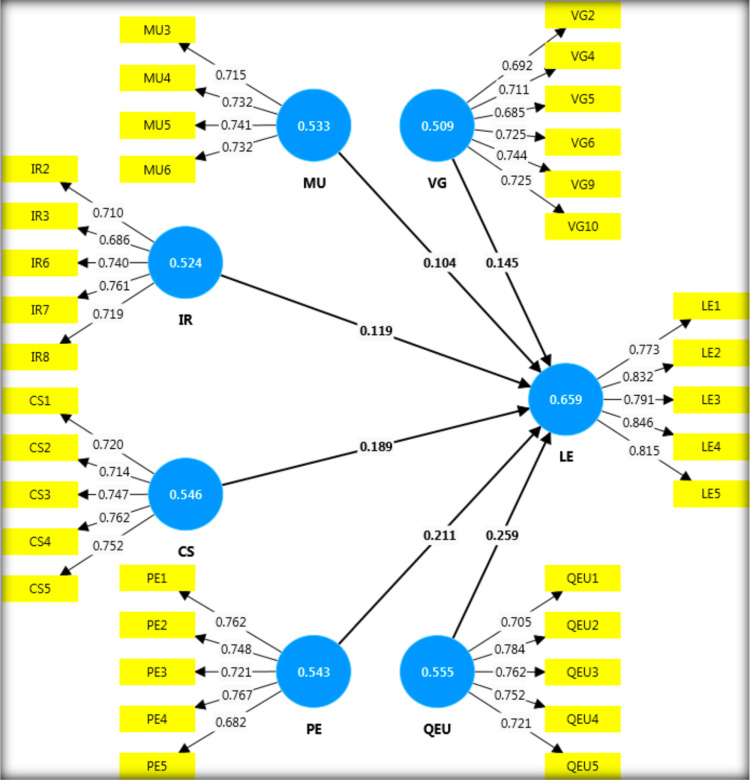
Measurement model.

The measurement model initially failed due to low Average Variance Extracted (AVE) values. To improve model fit, several low-loading indicators—specifically, VG1, VG3, VG7, VG8, VG11 (from Vision and Goal Setting), MU1, MU2, MU7, MU8 (from Management of the Unit), IR1, IR4, IR5, and IR9 (from Interpersonal Relationships)—were removed. This strategic removal of low-loading items increased the AVE values for the constructs, thereby achieving convergent validity. As shown in Table 2 and Fig 2, all constructs exceeded the minimum AVE threshold of 0.5, confirming convergent validity. Additionally, internal consistency reliability was assured with both Cronbach’s alpha and Composite Reliability (CR) values surpassing the 0.7 threshold. This methodological refinement aligns with best practices in scale validation, ensuring that the measurement model is both reliable and valid.

**Table 2:** Summary of results for convergent validity and internal consistency reliability.

Constructs	Scale	FL	AVE	CA	CR	rho_A
Vision and goal setting (VG)	VG2	0.725	0.509	0.808	0.862	0.809
	VG4	0.692				
	VG5	0.711				
	VG6	0.685				
	VG9	0.725				
	VG10	0.744				
Management of the unit (MU)	MU3	0.715	0.533	0.709	0.820	0.708
	MU4	0.732				
	MU5	0.741				
	MU6	0.732				
Interpersonal relationships (IR)	IR2	0.710	0.524	0.772	0.846	0.773
	IR3	0.686				
	IR6	0.740				
	IR7	0.761				
	IR8	0.719				
Communication skills (CS)	CS1	0.720	0.546	0.794	0.858	0.799
	CS2	0.714				
	CS3	0.747				
	CS4	0.762				
	CS5	0.752				
Research/Professional endeavors (PE)	PE1	0.762	0.543	0.789	0.855	0.792
	PE2	0.748				
	PE3	0.721				
	PE4	0.767				
	PE5	0.682				
Quality of the unit’s education (QEU)	QEU1	0.705	0.555	0.800	0.862	0.802
	QEU2	0.784				
	QEU3	0.762				
	QEU4	0.752				
	QEU5	0.721				
Leadership effectiveness (LE)	LE1	0.773	0.659	0.870	0.906	0.872
	LE2	0.832				
	LE3	0.791				
	LE4	0.846				
	LE5	0.815				

Concerning discriminant validity, the HTMT assessment results indicated that no construct had an HTMT value exceeding the 0.85 threshold (Table 3). Table 4 presents the Fornell-Larcker results, as recommended. Thus, the HTMT and Fornell-Larcker criteria verified the measurement model’s discriminant validity.

**Table 3:** Result for discriminant validity – HTMT.

	VG	MU	IR	CS	PE	QUE	LE
**VG**	–						
**MU**	0.690	–					
**IR**	0.693	0.750	–				
**CS**	0.637	0.617	0.754	–			
**PE**	0.721	0.729	0.734	0.824	–		
**QUE**	0.628	0.687	0.767	0.793	0.762	–	
**LE**	0.732	0.744	0.788	0.814	0.844	0.841	–

**Table 4:** Result for discriminant validity ((Fornell-Larcker).

	VG	MU	IR	CS	PE	QUE	LE
**VG**	**0.714**						
**MU**	0.527	**0.730**					
**IR**	0.553	0.559	**0.724**				
**CS**	0.518	0.472	0.592	**0.739**			
**PE**	0.581	0.548	0.575	0.657	**0.737**		
**QUE**	0.510	0.521	0.605	0.632	0.604	**0.745**	
**LE**	0.619	0.588	0.648	0.686	0.702	0.707	**0.812**

#### 4.1.2. Assessment of structural model and hypotheses testing.

The structural model is examined in the second phase of assessing the PLS-SEM findings [75]. The essential components that constitute the standard evaluation parameters for evaluating the structural model are standardized root mean square residual (SRMR), the Normed Fit Index (NFI), predictive relevance (Q^2^), collinearity assessment, coefficient of determination (R^2^), effect size (f^2^), and structural model path coefficients [77].

It is recommended that the SRMR value falls between 0.08 and 0.10, and the NFI value ranges from 0.70 to 0.90 to be considered acceptable [83]. The model’s SRMR and NFI were 0.066 and 0.711. So, the model fit was overall good. Since the scores were satisfactory, the model could be investigated further.

The subsequent analysis of the assessment model for predicting accuracy [84] was derived from the Q^2^ values [85]. Q^2^ values integrate in-explanatory power and out-of-sample prediction characteristics [86]. The prediction accuracy of the structural model depends on Q^2^ values greater than 0 for a dependent construct. Q² values indicate predictive relevance, with values exceeding 0, 0.25, and 0.50 representing small, medium, and large predictive relevance, respectively [76]. The Q^2^ value for leadership effectiveness is 0.667, which suggests a large predictive relevance.

The structural model uses OLS regressions for path coefficients. Hence, collinearity must be rigorously assessed to prevent bias in the regression results [77]. The construct’s variance inflation factor (VIF) value indicates collinearity. To avoid collinearity, the VIF value should be greater than 0.20 and less than 5 [87]. The data in Table 5 specifies that all VIF values fall within the recommended threshold.

**Table 5:** Result of collinearity assessment.

	VIF
VG -> LE	1.806
MU -> LE	1.752
IR -> LE	2.081
CS -> LE	2.207
PE -> LE	2.289
QEU -> LE	2.108

Next, the coefficient of determination (R^2^) is a widely used measure for assessing structural models. It represents the percentage of variance in the endogenous constructs explained by the correlated exogenous constructs [70]. Additionally, the R^2^ value measures the explanatory and sample predictive power of the model’s constructs [88,89]. R^2^ values are between 0 and 1, with higher values indicating stronger explanatory power. An R^2^ value of 0.75 indicates substantial explanatory power, 0.50 indicates moderate power, and 0.25 represents weak explanatory power [76,90]. The R^2^ coefficient is 0.689, as shown in Table 6. This indicates a satisfactory level of explanatory power, with the six exogenous constructs accounting for 68.9% of the variance in the endogenous construct.

**Table 6:** Results summary for R-square and f-square.

Exogenous constructs	f^2^	*P* values
Vision and goal setting	0.038	0.002
Management of the unit	0.020	0.037
Interpersonal relationships	0.022	0.028
Communication skills	0.052	0.001
Research/professional endeavors	0.063	0.002
Quality of the unit’s education	0.102	0.000
Endogenous construct	**R** ^**2**^	
Leadership effectiveness	0.689	

Furthermore, effect size, commonly represented as f^2^, measures the relative impact of exogenous constructs on endogenous construct. The f^2^ values of 0.02, 0.15, and 0.35 represent the small, medium, and large effects of an exogenous constructs on an endogenous construct [70,77]. As shown in Table 6, vision and goal setting, management of the unit, interpersonal relationships, communication skills, and research/professional endeavors exhibit small but statistically significant effect sizes, while the quality of the unit’s education shows a medium and significant effect size, reinforcing its importance in the model.

**Fig 3:**
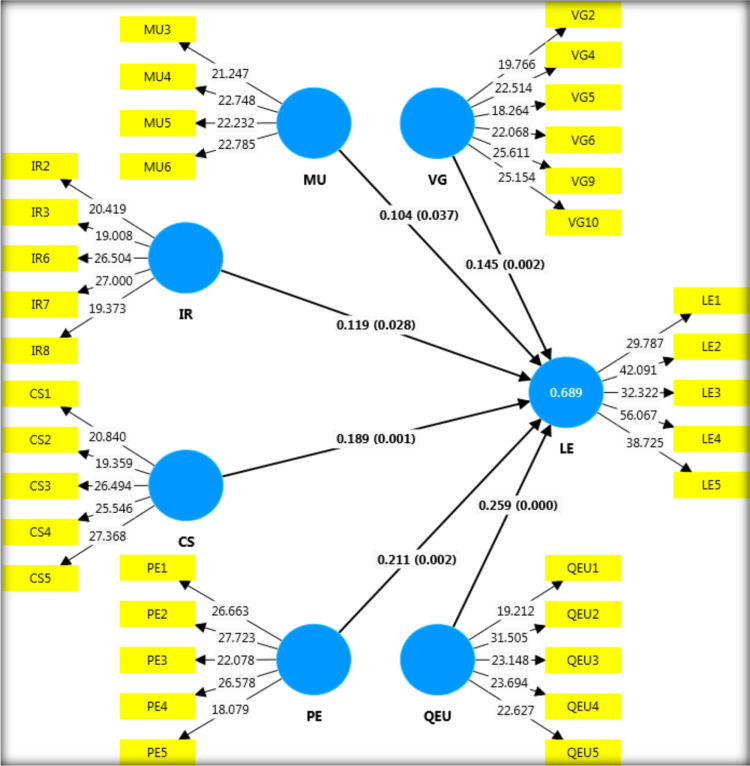
Structural Model.

Structural model evaluation uses the stated hypothesis to examine the path between constructs. Following [70], we calculated the sample’s standard error and t-statistics and assessed the path coefficients using bootstrapping with 5000 subsamples, a two-tailed approach, and a significance level of 0.05. The statistical significance of all hypothesized relationships is shown in Table 7 and Fig 3. However, vision and goal setting (β =  0.145, t =  3.135, p =  0.002), management of the unit (β =  0.104, t =  2.091, p =  0.037), and interpersonal relationships (β =  0.119, t =  2.200, p =  0.028) have a positive and significant effect on academic leadership effectiveness supporting H_1_, H_2,_ and H_3_, respectively. Similarly, hypotheses H4, H5, and H6 are supported by the strong positive impacts of communication skills (β =  0.189, t =  3.182, p =  0.001), research/professional endeavors (β =  0.211, t =  3.176, p =  0.002), and quality of the unit’s education (β =  0.259, t =  4.453, p =  0.000) on leadership effectiveness.

**Table 7:** Hypothesis testing result.

Hypothesis	Paths	Path coefficient (O)	Standard Deviation (SD)	T statistics (|O/S.D|)	*P* values	Result
***H***_***1***_	Vision and goal setting -> Leadership effectiveness	0.145	0.046	3.135	0.002	**Supported**
***H***_***2***_	Management of the unit -> Leadership effectiveness	0.104	0.050	2.091	0.037	**Supported**
***H***_***3***_	Interpersonal relationships -> Leadership effectiveness	0.119	0.054	2.200	0.028	**Supported**
***H***_***4***_	Communication skills -> Leadership effectiveness	0.189	0.059	3.182	0.001	**Supported**
***H***_***5***_	Research/professional endeavors -> Leadership effectiveness	0.211	0.066	3.176	0.002	**Supported**
***H***_***6***_	Quality of the unit’s education -> Leadership effectiveness	0.259	0.058	4.453	0.000	**Supported**

### 4.2. Fuzzy-set qualitative comparative analysis (fsQCA) results

Further analysis was conducted using the fsQCA tool to determine how the various components combined to get a certain outcome. This study’s antecedent conditions were six independent variables: VG, MU, IR, CS, PE, and QEU, while the outcome variable was LE. The three key phases in the data analysis are data calibration, constructing a truth table, and causal conditions analysis [91].

#### 4.2.1. Calibration.

The calibration process in fsQCA converts raw scores into a 0-1 scale. The present study used latent variable scores for calibration because they more accurately represent unobservable constructs [73,92], therefore improving the accuracy of further fsQCA evaluations. This was accomplished by taking an average of the associated indicators and using it to generate an index for each construct. Fuzzy sets must be created from ordinary data by setting Likert scale values to full membership (fuzzy score =  0.95), crossover point (fuzzy score =  0.5), and full non-membership (fuzzy score =  0.05).

Variables were converted into calibrated sets, with 4 representing complete membership, 3 representing the crossover point, and 2 representing full non-membership [77]. As recommended by Fiss [93], this study adds 0.001 to scores exactly equal to 0.50 to ensure reliability and prevent complications. This prevents cases close to the crossover point from being excluded from analysis. In fsQCA V. 4.1, the calibration procedure was automated. Table 8 illustrates the outcomes of this transformation together with descriptive statistics of the variables, which serve as the fundamental fuzzy sets for further assessment of causal conditions and outcomes.

**Table 8:** Calibration threshold for measures and descriptive statistics of the variables.

Antecedent conditions	Fuzzy set calibration			Descriptive statistics				
	Complete affiliated point	Intersection point	Complete non-affiliated point	Mean	Std. Dev.	Min	Max	N
VG	4	3	2	3.52	0.62	1.7	5	318
MU	4	3	2	3.64	0.60	2.3	5	318
IR	4	3	2	3.69	0.61	2	5	318
CS	4	3	2	3.58	0.69	1.2	5	318
PE	4	3	2	3.48	0.67	1.2	5	318
QEU	4	3	2	3.56	0.68	1.2	5	318
LE	4	3	2	3.47	0.71	1	5	318

#### 4.2.2. Necessity condition analysis.

A crucial part of fsQCA is the examination of sufficient conditions, but before looking into that, it is required to determine the necessary conditions [91]. The necessary conditions analysis in fsQCA is employed to confirm whether the antecedent conditions are necessary for the outcome. This study inspects all conditions, both present and absent, with a focus on the presence or absence of LE as the outcome variable. It identifies six preconditions - VG, MU, IR, CS, PE, and QEU - affecting LE. According to Ragin [91], a variable is usually deemed “necessary” for the outcome if its consistency is greater than 0.9.

It is evident from Table 9 that all the conditions have sufficient consistency scores except VG, leading to impacting high leadership effectiveness since their scores for consistency are above the threshold level. Hence, the result reveals that MU, IR, CS, PE, and QEU are considered essential to ensure the deans’ high leadership effectiveness. Additionally, necessary conditions in fsQCA offer a vital understanding of the fundamental mechanisms of causal relationships. The interrelated nature of these conditions implies that their combined impact should be explored in more detail.

**Table 9:** Causal necessary condition test.

Antecedent conditions tested	Leadership effectiveness (LE)	
**Consistency**	**Coverage**
Vision and goal setting ( ~ VG)	0.898 (0.264)	0.860 (0.707)
Management of the unit ( ~ MU)	0.922 (0.229)	0.835 (0.731)
Interpersonal relationships ( ~ IR)	0.942 (0.205)	0.831 (0.727)
Communication skills ( ~ CS)	0.916 (0.233)	0.857 (0.670)
Research/professional endeavors ( ~ PE)	0.904 (0.270)	0.890 (0.674)
Quality of the unit’s education ( ~ QEU)	0.924 (0.249)	0.877 (0.687)

**Note:** “~” denotes the negation or absence of a causal condition.

#### 4.2.3. Sufficient condition analysis.

After the analysis of necessity, the analysis of sufficient conditions was conducted to find the various causal configurations that result in leadership effectiveness. For the examination of sufficient conditions, a truth table with 2^k^ rows was constructed. Each row represents a combination of six factors, along with the frequency and consistency of each combination, and k represents the number of outcome factors [91]. To ensure that 75% of the reserved samples are remained and that the study’s sample size is considered to be large (>150 cases), a minimum frequency threshold of 3 was established [94,95]. The raw consistency standard was set at 0.8 to delineate the minimum impact of the antecedent conditions on the outcome [96].

Complex, intermediate, and parsimonious solutions were reported in the standard analyses generated by the fsQCA truth table [91]. The complex solution lacked explanatory value, while the parsimonious and intermediate solutions successfully made a distinction between the core and peripheral conditions [94]. Table 10 presents the results of the fsQCA findings, which identify six unique solutions that contribute to high leadership effectiveness (LE) in the context of Bangladeshi public universities. Table 10 also demonstrates that the consistency and coverage of all configurations are above 0.8 and 0.2, respectively [92].

**Table 10:** Sufficient solutions for high leadership effectiveness.

Antecedent conditions	Solutions for high leadership effectiveness					
	S-1	S-2	S-3	S-4	S-5	S-6
VG	X	X	X			X
MU	0			0	0	0
IR	X	X	X	X	X	
CS		0	0	0		0
PE		0			0	0
QEU			X	X	X	X
**Consistency**	0.910	0.949	0.947	0.937	0.951	0.964
**Raw coverage**	0.822	0.774	0.779	0.795	0.790	0.738
**Unique coverage**	0.028	0.003	0.004	0.009	0.005	0.014
**Solution consistency**	0.920					
**Solution coverage**	0.890					

Notes: X =  Presence of core causal condition, 0 =  Presence of peripheral causal condition,

Blank cell =  Presence or absence of condition doesn’t matter.

The result indicates that all six solutions were sufficient for leadership effectiveness, as the consistency values exceeded 0.8. The solutions are: Solution-1: VG *  MU *  IR; Solution-2: VG *  IR *  CS *  PE; Solution-3: VG *  IR *  CS *  QEU; Solution-4: MU *  IR *  CS *  QEU; Solution-5: MU *  IR *  PE *  QEU; and Solution-6: VG *  MU *  CS *  PE *  QEU. Findings from Solutions 1 indicate that leadership effectiveness can be achieved through a combination of VG, MU, and IR and CS, PE, and QEU are irrelevant. According to solution 2, Leadership effectiveness can be attained by combining VG, IR, CS, and PE, while MU and QEU have insignificant roles. These findings from solution 1 and 2 emphasize the crucial role of VG and IR in attaining high LE (core conditions). The sufficient configurations ensure high LE with a raw coverage of 82.2% and 77.4% of the cases, respectively. Also, these solutions exhibit a high level of consistency with scores of 0.910 and 0.949. Next, a high level of LE can be attained with a combined recipe of VG, IR, CS, and QEU, as reported by 77.9% of the cases in Solutions 3 where MU and PE are not important.

This solution has a high consistency score of 0.947. Solution 4 further proposes that MU and IR, when combined with CS and QEU, are sufficient to achieve a high level of LE while VG and PE are inconsequential according to 79.5% of the cases. This solution has a high consistency score of 0.937. Solution 5 then highlights the significant role of IR and QEU in achieving high LE combined with MU and PE. Lastly, solution 6 underscores that the combination of VG and QEU can still achieve high LE even with a low MU, CS, and PE level. Notably, Solution 1 is the most effective solution for achieving high LE, as evidenced by its high raw coverage of 0.822. This implies that it applies to a wide range of cases. In addition, the combinations of solutions associated with high leadership effectiveness accounted for a significant portion of the overall solution coverage, which is 89%. The six configurations could likely explain 92% of the cases with adequate explanatory power, as the overall solution consistency was 0.920.

### 4.3. Necessary condition analysis (NCA) results

**Fig 4:**
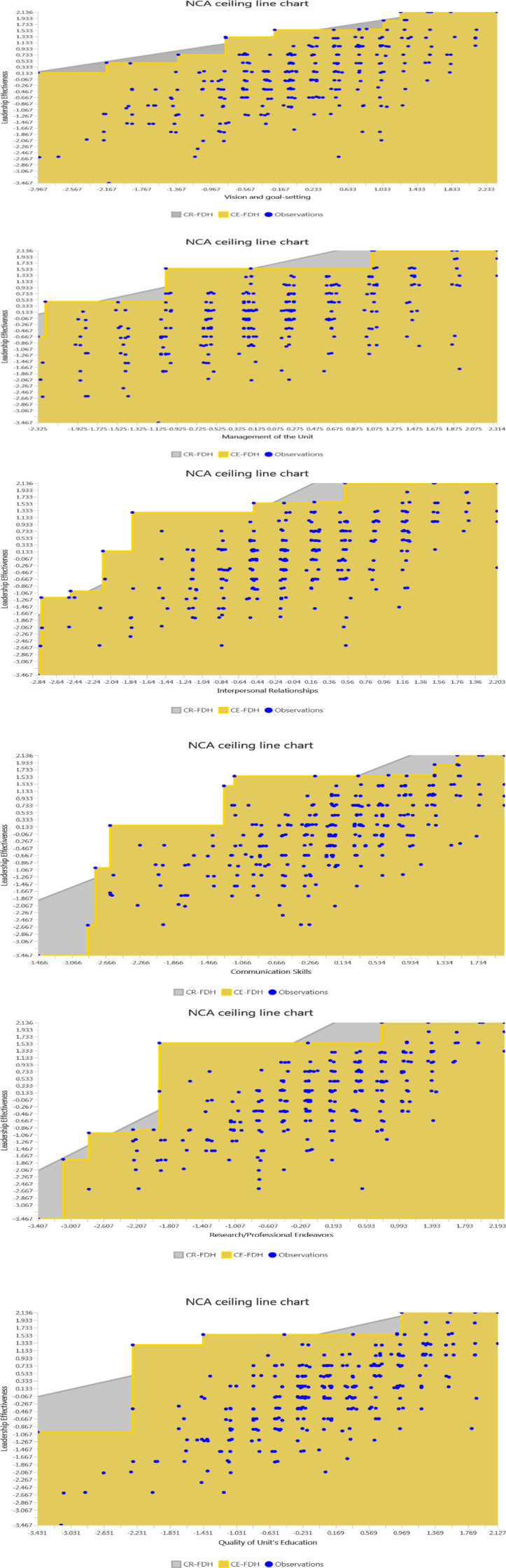
Scatter plot of the predictor variables and the outcome.

Necessary Condition Analysis (NCA) was performed to supplement the PLS-SEM further to examine the relationships between predictor variables and the outcome. Three main criteria used to determine the necessary conditions [97]. Firstly, the predictor-outcome variable relationship needs to be theoretically justified. Secondly, the necessary condition’s effect size should exceed zero to be significant [98]. Thirdly, evaluation of the conditions against the null hypotheses to avoid making Type 1 errors and giving false results. To accomplish this, a bootstrapping approach can be implemented, which involves employing a permutation test to evaluate the necessary conditions against the outcome. If the relationship is to be considered significant, it must have a low p-value, such as p < .05. The Cartesian coordinate system was used to initiate NCA, with the latent variable scores from the PLS-SEM analysis serving as the starting point. Different ceiling lines can be selected. The proposed Ceiling Regression-Free Disposal Hull (CR-FDH) line was performed in this study (Fig 4), which is ideal for continuous or discrete data with numerous levels [99].

After that, the effect sizes (d) of the latent variable scores were subsequently evaluated, and a recommended random sample size of 10,000 was used to figure out if the results were statistically significant [97,100]. In Table 11, the findings revealed that each condition was necessary for leadership effectiveness, as they demonstrated effect sizes that exceeded zero. The effect size of communication skills was the most significant (d =  0.290, p < .05), followed by research/professional endeavors (d =  0.240, p < .05). This suggests a medium effect size range (0.1 ≤  d ≤  0.3) [96]. Likewise, interpersonal relationships (d =  0.207, p < .05), quality of the unit’s education (d =  0.164, p < .05), vision and goal setting (d =  0.152, p < .05) and management of the unit (d =  0.121, p < .05) were also determined as necessary conditions having medium effect sizes.

**Table 11:** Effect sizes (d) and significance in NCA.

	Leadership effectiveness (LE)	
	CR-FDH	P-value
VG	0.152	0.000**
MU	0.121	0.000**
IR	0.207	0.000**
CS	0.290	0.000**
PE	0.240	0.000**
QUE	0.164	0.000**

Note: “**” means the effect size is medium.

A bottleneck table (Table 13) was developed at the final stage to obtain a more in-depth understanding of the essential predictors’ levels for ensuring leadership effectiveness. Table 12 demonstrates the minimum values (in percentage) needed for predictor variables (VG, MU, IR, CS, PE, and QEU) in the subsequent columns, corresponding to each desired level of the outcome variable (LE) listed in the first column. As articulated, the minimum requirements for achieving leadership effectiveness of 80% were: 14.78% for VG, 20.75% for MU, 23.27% for IR, 33.96% for CS, 16.98% for PE, and 10.6% for QEU. However, as the level of LE (100%) increases, the required percentages of predictor variables also increase. These values imply that deans are unlikely to exhibit high leadership effectiveness unless the specified threshold values are not attained. More importantly, this finding consistently conforms to the results obtained from both PLS-SEM and fsQCA analyses.

**Table 12:** Bottleneck table in NCA (%).

LE	VS	MU	IR	CS	PE	QUE
0%	N	N	N	N	N	N
10%	N	N	N	N	N	N
20%	N	N	N	N	N	N
30%	N	N	N	0.31	0.31	N
40%	N	N	2.20	0.94	1.26	N
50%	N	N	3.77	3.14	3.14	N
60%	N	N	5.66	7.55	6.29	N
70%	1.26	5.66	13.21	16.98	11.63	1.57
80%	14.78	20.75	23.27	33.96	16.98	10.06
90%	63.21	50.00	36.79	61.95	36.48	48.74
100%	96.23	79.24	57.55	84.59	58.49	85.53

Note: “NN” indicates that it is not necessary. The shaded area highlights the minimum percentage of each predictor variable required for high leadership effectiveness.

**Table 13:** Key findings from the combined analytical approach.

Key constructs	PLS-SEM findings	fsQCA findings	NCA findings
VG	…is a significant contributor to leadership effectiveness.	… is not necessary but sufficient condition to ensure high LE.	… is a necessary condition for high LE (> 14.78%) and has medium effect.
MU	…is a significant contributor to leadership effectiveness.	… is both necessary and sufficient condition to ensure high LE.	… is a necessary condition for high LE (20.75%) and has medium effect (lowest among all conditions).
IR	…is a significant contributor to leadership effectiveness.	… is both necessary and sufficient condition to ensure high LE.	… is a necessary condition for high LE (> 23.27%) and has medium effect.
CS	…is a significant contributor to leadership effectiveness.	… is both necessary and sufficient condition to ensure high LE.	… is a necessary condition for high LE (33.96%) and has medium effect (highest among all conditions).
PE	…is a significant contributor to leadership effectiveness.	… is both necessary and sufficient to condition ensure high LE.	… is a necessary condition for high LE (16.98%) and has a medium effect.
QEU	is a significant contributor to leadership effectiveness.	… is both necessary and sufficient condition to ensure high LE.	… is a necessary condition for high LE (> 10.06%) and has a medium effect.

## 5. Discussion and Implications

### 5.1. Discussion of key findings

This study conducted an empirical investigation into faculty members’ perceptions regarding the effectiveness of deans’ leadership in public universities in Bangladesh. This study developed and examined a model in which vision and goal setting, management of the unit, interpersonal relationships, communication skills, research/professional endeavors, and quality of education in the unit are examined as contributors to leadership effectiveness. A combined approach of the PLS-SEM, fsQCA, and NCA methods is utilized in this research.

The PLS-SEM results indicated that VG, MU, IR, CS, PE, and QEU positively influence the dean’s leadership effectiveness (Table 7). This aligns with the findings of Sehgal et al. [101] which suggests that these are likely to enhance leadership effectiveness significantly. Furthermore, the findings are compatible with previous research that presents a framework for evaluating the academic’s leadership effectiveness according to these six leadership domains or roles [102]. Academic leadership effectiveness is not just about identifying organizational elements but also ensuring all staff, regardless of position, are engaged and aligned with elements like VG, MU, and CS [103]. Nevertheless, there is a strong connection between positive interpersonal relationships and pedagogical leadership, which can enhance the educational environment and improve teacher performance and student outcomes [104]. However, one study does not directly support the connection between professional endeavor (PE) and leadership effectiveness of teachers. The study suggests that principal leadership, emphasizing values like integrity and trustworthiness, plays a key role in developing teachers and staff, ultimately influencing academic leadership, as found in this paper [105]. This study found a significant connection between quality education and academic leadership, which is further emphasized by another study, underlining that improving academic leadership effectiveness cannot be achieved without including quality education (SDG-4) [106,107].

While the findings from the PLS-SEM provide insights into the overall impact of antecedents on outcomes, fsQCA offers various particular combinations of antecedent conditions that are sufficient. Thus, fsQCA indicated six primary configurations which can significantly influence high LE (Table 11). The findings validated the conditions as necessary and sufficient for achieving high LE. According to solutions 1 and 2, the combination of VG and IR (core requirements), along with MU or with CS and PE, could result in achieving high leadership effectiveness. Solution 3 demonstrated that when VG and IR are aligned with CS and QEU, it could lead to a high level of LE. Based on solutions 4 and 5, it was found that combining MU, IR, and QEU with CS or PE might result in high LE. Additionally, solution 6 showed that the combination of VG, MU, CS, PE, and QEU could also lead to high LE. Core conditions for high LE are VG, IR, and QEU, as evidenced by the fsQCA results.

In addition, the PLS-SEM findings (H1) are consistent with the fact that VG is a condition in four of the six configurations (solutions 1, 2, 3, and 6). Likewise, the PLS-SEM results (H3) are also consistent with the fact that IR is an essential prerequisite in five of the six solutions. Moreover, the PLS-SEM result (H6) is validated by the core condition of QEU in solutions 3-6, which results in a high LE. Apart from these, the PLS-SEM results (H2, H4, and H5) are further supported by the identification of MU, CS, and PE as significant factors (peripheral conditions) that ensure high LE.

Although fsQCA evaluated the empirical value of sufficient combinations of conditions for high LE, NCA offered a better comprehension of how the predictor variables confined the outcome variable. NCA results confirmed that all six conditions are essential for achieving the desired outcome, which is consistent with the PLS-SEM and fsQCA findings. Notably, VG was found to be unnecessary according to necessity results in fsQCA but it is necessary according to NCA results. Additionally, they have medium-size influences on LE. In particular, LE is most significantly influenced by communication skills. Furthermore, the NCA bottleneck analysis (Table 12) illustrated the minimum level of predictors necessary to achieve a high level of outcome. For a minimum of 80% of LE to be achieved, the required percentages of VG, MU, IR, CS, PE, and QEU must be 14.78%, 20.75%, 23.27%, 33.96%, 16.98%, and 10.06%, respectively. It has also been discovered that the minimum required percentages of the predictor variables increase as the level of LE increases.

Table 13 shows the combined results of the three analytical approaches, which offer an outline for comparing the results of each method and deriving the final insights. This framework helps identify significant links between hypothesized predictors and the outcome, ascertain the presence of necessary conditions, and evaluate cases with alternative causal configurations. It allows for the classification and description of complexities, illustrates variations, and enhances the explanatory power of the examined phenomenon.

### 5.2. Theoretical implications

This study contributes to leadership theory by utilizing a combination of PLS-SEM, fsQCA, and NCA methods, demonstrating that a multi-method approach can provide a more comprehensive understanding of leadership effectiveness. By integrating these methods, the study highlights the existence of multiple pathways (or configurations) that can lead to effective leadership, thereby supporting the concept of equifinality—where different combinations of factors can achieve the same outcome. This finding encourages future research to move beyond single-method studies, suggesting that employing diverse analytical techniques can capture the complexities of leadership dynamics more effectively.

The present study also extends existing leadership theories to new cultural and institutional contexts by investigating academic leadership in public universities in Bangladesh. This contextualized insight enriches the global discourse on leadership by highlighting the need for theories that are adaptable to diverse environments. The study also provides clarity on the roles of necessary and sufficient conditions for leadership success, offering theoretical frameworks for identifying which elements must always be present and which combinations can effectively achieve desired outcomes. This distinction is vital for refining theoretical models and guiding future research to focus on the critical factors that drive leadership effectiveness.

### 5.3. Managerial implications

From a managerial perspective, this study gives perceptions of university leaders and policymakers who seek to enhance leadership effectiveness in academic settings. Universities should recognize that multiple combinations of attributes can lead to effective leadership. This insight calls for the design of flexible and customized training programs that address the diverse needs of academic leaders. These programs should incorporate various methods to develop a broad range of competencies, including VG, MU, IR, CS, PE, and QUE, ensuring that leaders are well-rounded and adaptable to different challenges.

Moreover, institutions should strategically allocate resources to strengthen both core and supporting leadership factors identified in the study. This could involve investing in professional development opportunities. The study also suggests implementing a comprehensive evaluation framework that integrates multiple methods to gain a deeper understanding of leadership effectiveness. Such an approach would allow university administrators to more accurately assess leadership performance, guiding more effective decision-making and policy development. The data-driven insights provided by this research emphasize the importance of continuous monitoring and adaptation of leadership strategies.

By leveraging empirical evidence, institutions can refine their leadership approaches to better meet their unique needs. This practice helps ensure that leadership strategies remain aligned with institutional goals and are responsive to evolving challenges, fostering a more effective and resilient academic leadership environment.

## 6. Conclusions

This study provides a comprehensive examination of the perceptions of leadership effectiveness among deans in public universities in Bangladesh, focusing on six key leadership constructs: VG, MU, IR, CS, PE, and QEU. By employing a combined approach of PLS-SEM, fsQCA, and NCA methods, the study offers a nuanced understanding of how these factors contribute to effective leadership. The PLS-SEM analysis demonstrates that all six constructs significantly impact leadership effectiveness, while fsQCA uncovers six distinct configurations of these variables that can lead to high effectiveness, with combinations involving VG, MU, and IR proving particularly effective (Based on raw coverage). Additionally, the NCA method highlights the necessity of all six factors and identifies specific bottlenecks that must be addressed to achieve high leadership effectiveness. The integrated approach of these three methods confirms the existence of complex, asymmetric relationships among the variables, providing deeper insights into the mechanisms of effective leadership in academic settings.

While the study offers valuable insights, it also has certain limitations that suggest avenues for future research. The focus on faculty members from eight public universities in Bangladesh limits the generalizability of the findings. Future studies could expand the sample size and include a broader range of universities to enhance the applicability of the results. The cross-sectional design also restricts the ability to capture the dynamic nature of leadership over time, pointing to the need for longitudinal studies. Additionally, relying solely on faculty perceptions of leadership effectiveness presents a limited perspective; future research could incorporate self-assessments from deans as well as evaluations from their superiors, subordinates, and students. Exploring leadership effectiveness in different contexts, such as comparing public and private universities, and using alternative instruments like the Multifactor Leadership Questionnaire (MLQ), could provide further insights. Despite its methodological rigor, this study recognizes that there are other analytical methods that may offer deeper explanations of the research phenomena, encouraging future researchers to consider diverse approaches.Top of Form

## Supporting information

S1 Table Measures of the study. (DOCX)

S1 Checklist PLOSOne Human Subjects Research Checklist. (DOCX)

S1 Data Data Set PONE-D-24-39175R2 - [EMID17dc8330e1f4f063]. (CSV)
