# Supplementary material for: Unveiling perceptions on academic leadership effectiveness: PLS-SEM, FSQCA, and NCA approaches
Source: PLoS One. 2025 Apr 14;20(4):e0320723. doi: 10.1371/journal.pone.0320723 (PMC11996076; doi:10.1371/journal.pone.0320723)
Supplement: S1 Table — (DOCX) [file pone.0320723.s001.docx]

# **7. Appendix: Supporting information**

**S1 Table. Measures of the study.**

| Constructs/Indicators | Indicators details |
| --- | --- |
| Vision and Goal Setting |  |
| VG2 | To what extent does your leader encourage ideas and creativity? |
| VG4 | To what extent does he/she demonstrate vision and long-range planning? |
| VG5 | To what extent does he/she emphasize teaching excellence appropriately? |
| VG6 | To what extent does he/she emphasize research excellence appropriately? |
| VG9 | To what extent does he/she encourage faculty development? |
| VG10 | To what extent does he/she encourage curriculum/program development? |
| Management of the Unit |  |
| MU3 | To what extent does he/she manage changes constructively? |
| MU4 | To what extent does he/she delegate work effectively? |
| MU5 | To what extent does he/she handle administrative tasks in a timely manner? |
| MU6 | To what extent is he/she an effective problem solver? |
| Interpersonal Relationships |  |
| IR2 | To what extent does he/she treat individuals fairly and with respect? |
| IR3 | To what extent does he/she maintain positive and productive relationships within the unit? |
| IR6 | To what extent does he/she demonstrate sensitivity to the career and mentoring needs of unit members? |
| IR7 | To what extent he/she is accessible to faculty and staff within the unit? |
| IR8 | To what extent does he/she demonstrate an understanding of the needs and concerns of students? |
| Communication Skills |  |
| CS1 | To what extent does your leader listen to and communicate with unit members? |
| CS2 | To what extent does he/she listen to and communicate with external constituencies? |
| CS3 | To what extent does he/she effectively represent the unit and its members to the rest of the university? |
| CS4 | To what extent does he/she effectively communicate the unit's priorities to the upper-level administration? |
| CS5 | To what extent does he/she produce clear reports and correspondence? |
| Research/Professional Endeavors |  |
| PE1 | To what extent does your leader maintain an active research/scholarly agenda? |
| PE2 | To what extent does he/she pursue professional growth opportunities? |
| PE3 | To what extent does he/she engage in effective teaching? |
| PE4 | To what extent does he/she contribute his/her services to professional organizations? |
| PE5 | To what extent does he/she contribute his/her services to community and campus projects? |
| Quality of Education in the Unit |  |
| QEU1 | To what extent does your leader advance the unit's graduate programs effectively? |
| QEU2 | To what extent does he/she advocate appropriate curriculum offerings? |
| QEU3 | To what extent does he/she handle external accreditation reviews effectively? |
| QEU4 | To what extent does he/she recruit new personnel and/or promote recruitment skillfully? |
| QEU5 | To what extent does your leader demonstrate a commitment to ensuring a fair tenure and promotion process? |
| Leadership Effectiveness |  |
| LE1 | To what extent does your leader meet managerial performance standards? |
| LE2 | To what extent does he/she have overall leadership success? |
| LE3 | To what extent he/she is successful in comparison to his/her managerial peers? |
| LE4 | To what extent does he/she perform as a role model? |
| LE5 | To what extent he/she is overall effective as a leader? |
